# Thiamin stimulates growth, yield quality and key biochemical processes of cauliflower (*Brassica oleracea* L. var. Botrytis) under arid conditions

**DOI:** 10.1371/journal.pone.0266372

**Published:** 2022-05-25

**Authors:** Munifa Jabeen, Nudrat Aisha Akram, Muhammad Ashraf, Anshika Tyagi, Mohamed A. El-Sheikh, Parvaiz Ahmad

**Affiliations:** 1 Department of Botany, Government College University, Faisalabad, Pakistan; 2 University of Lahore, Lahore, Pakistan; 3 Department of Biotechnology, Yeungnam University, Gyeongsan, South Korea; 4 Botany and Microbiology Department, College of Science, King Saud University, Riyadh, Saudi Arabia; 5 Department of Botany, Govt. Degree College, Pulwama, Srinagar, Jammu and Kashmir, India; National University of Kaohsiung, TAIWAN

## Abstract

Thiamin is a crucial vitamin with a vast variety of anti-oxidative and physiological roles in plants subjected to abiotic stresses. We examined the efficiency of foliar-applied thiamin (50 and 100 mM) on growth, yield quality and key-biochemical characteristics of two cultivars (FD1 and FD3) of cauliflower (*Brassica oleracea* L.) under water-deficit stress. Water stress at the rate of 50% field capacity (F.C.) markedly decreased the plant biomass, leaf total phenolics and ascorbic acid (AsA) contents. In contrast, drought-induced increase was noted in the leaf [hydrogen peroxide (H_2_O_2_), AsA, proline, malondialdehyde (MDA), glycinebetaine (GB), total soluble proteins and oxidative defense system in terms of high activities of peroxidase (POD), and catalase (CAT) enzymes] and the inflorescence (total phenolics, proline, GB, MDA, H_2_O_2_, and activities of SOD and CAT enzymes) characteristics of cauliflower. However, foliar-applied thiamin significantly improved growth and physio-biochemical attributes except leaf and inflorescence MDA and H_2_O_2_ contents of both cauliflower cultivars under water stress. Overall, application of thiamin enhanced the plant growth may be associated with suppressed reactive oxygen species (ROS) and upregulated antioxidants defense system of cauliflower.

## Introduction

Abiotic stress factors including drought, salinity and heat affect different characteristics of the plants from seed germination to the adult stage [1]. Of these environmental challenges, water scarcity is the most serious threat to crop production which affects crop yield and quality [2, 3]. Generally, drought stress disturbs several plant activities including leaf gas exchange, carbon assimilation rate, cell turgor, oxidative defense system; all these changes lead to decrease in crop yield [4, 5]. Crop sensitivity or tolerance to water stress is a complex situation and is linked with activities of key enzymes, pattern of nutrient uptake, and defense mechanism thereby leading to reduce crop yield [6, 7].

Photosynthetic machinery of plant is affected due to drought-induced closure of stomata leading to a marked reduction in uptake of carbon dioxide (CO_2_) [8]. High production of reactive oxygen species (ROS) is a primary response of biotic and abiotic stresses and under such circumstances, plant’s oxidative defense system is triggered to sustain growth [9–14]. Reactive oxygen species including alkoxy radicals, hydrogen peroxide, singlet-oxygen, hydroxyl radical and superoxide can destruct cell membrane, lipids, proteins and macromolecules such as DNA and RNA but the extent of such damage is markedly high in stress sensitive species [15, 16].

Thiamin, a vitamin B_1_, plays an essential role as a co-enzyme in several metabolic processes including pentose phosphate pathway, tricarboxylic acid cycle, formation of branched amino acids and glycolysis in cellular organisms [17, 18]. It is recognized that high concentration of thiamin in seeds, roots and leaves of plants is helpful in promoting crop growth and productivity under stress situations [17, 19–21]. Thiamin is involved in altering the negative impacts of environmental stresses in microorganisms as well as plants [22, 23]. Application of thiamin decrease the negative effects of water stress on several plants [19, 24]. For example, foliar-applied thiamin considerably improved the tolerance of *Trifolium repens* to drought stress (80% and 60% field capacities). They observed that 100 mM thiamin was found effective in improving growth of two white clover cultivars which can be associated with thiamin-induced increase in phenolic contents and chlorophyll pigments [19]. Thiamin is believed to ameliorate water stress induced adversaries by improving oxidative defense system of plants. Several studies suggested that thiamin is effective as an antioxidant. Endogenous thiamin level increases by its foliar application or the activation of transketolase enzymes could increase antioxidant defense system under water stress. Moreover, progressive drought stress induces oxidative stress in plants [19]. Interestingly, plants have the capability to produce enzymatic and non-enzymatic antioxidants including CAT, SOD, POD, phenolics and ascorbic acid to maintain normal metabolic activities under drought stress. Environmental features including temperature, light, nutrition and water supply may impact the concentration of bioactive substances and chemical composition in vegetables, which directly disturbs antioxidant potential [36]. They recommended that the usage of thiamin defends seedlings against reactive oxygen species by increasing oxidative defense system [19, 25].

Cauliflower (*Brassica oleracea* L.) is a very common vegetable of sub-temperate region of the world [26]. However, its production is hampered under drought stress [27]. Cauliflower is much susceptible to drought stress at different growth stages including seedling, vegetative and reproductive, responding to drought with decreased/premature heading and growth of leaves. So, growth, production of dry matter and yield are badly affected due to drought stress. The optimum level of irrigation is essential for attaining maximum yield production worldwide [27]. This is mainly factual for vegetables including cauliflower, in which drought stress negatively affects quality e.g., color, inflorescence size and nutritional value. Therefore, the present study was conducted to assess the role of foliar-applied thiamin in ameliorating the adverse effects of water shortage on growth, quality of inflorescence and key physio-biochemical processes of both cultivars (FD1 and FD3) of cauliflower.

## Materials and methods

An experiment was laid out to study the interactive effect of foliar-applied thiamin and drought stress on physiological, biochemical and morphological attributes of two cultivars (FD1 and FD3) of cauliflower (*Brassica oleracea* L. var. Botrytis). During the study, relative humidity 73%, average temperature 33.7 °C, and light period 14.2 h were noted. Ten seeds of each cauliflower cultivar were sown per pot. The experimental soil was sandy loam, having pH 8.6, EC, 3.83 dS/m, organic matter 0.90%, K 400 mg L^-1^, P 5.8 mg L^-1^ and saturation percentage, 34%. After germination, thinning of plants was done to maintain five plants of uniform size with four replications of each treatment. Three levels of water stress [control (100%), 75% and 50% field capacities (F.C.)] were initiated after fifteen days of germination by regularly weighing the pots on the basis of required F.C. Water content of the pots was monitored daily on the basis of volume to maintained the soil water content at 100%, 75% and 50% F.C. The soil water content/F.C. were measured on the basis of percentage of moisture contents. The required levels of drought stress treatments were achieved after 13 days of initiation of drought stress and continued up to four weeks. After it, three levels of thiamin [0 (water spray as a control), 50 and 100 mM] were applied to the plants with the help of a hand pump. Initially, a stock solution of 100 mM concentration of thiamin was prepared using distilled water and further diluted up to 50 mM. About 400 mL of each concentration was prepared and added 0.1% Tween-20 as a surfactant to it before foliar spray. Approximately, 25 mL of each level of thiamin was applied per pot. After 15 days of thiamin application, two plants were uprooted from each pot, and lengths as well as fresh and dry weights of roots and shoots were recorded. The remaining three plants were allowed to grow till the adult stage to record yield. Moreover, fresh leaves and inflorescence were kept at -20 ֯C to analyze the following physio-biochemical attributes:

### Chlorophyll contents

Fresh leaf (0.5 g) was extracted in 80% acetone and the mixture kept for overnight. The protocol of Arnon [28] was employed to quantify chlorophyll contents. The absorbance was determined at 645 and 663 nm using a spectrophotometer.

### Relative Water Contents (RWC)

Following the protocol of Jones and Turner [29], the fresh weight of leaves was recorded soon after harvesting. Then, the plant leaves were placed in a water container for three hours and the turgidity of all samples was measured. After that, the leaf samples were placed in an oven for 72 h and their dry weights determined.

### Relative Membrane Permeability (RMP)

A fresh leaf (0.5 g) was chopped and kept in distilled water (10 mL). Then EC_0_ of each extract was determined after two hours. The samples were kept for overnight and EC_1_ recorded. Thereafter, the samples were kept in an autoclave at 120°C for 25 min and EC_2_ recorded. The relative membrane permeability was determined following Yang et al. [30].

### Free proline contents

Following Bates et al. [31] proline content was measured by extracting 500 mg fresh leaf and inflorescence samples in 10 mL (3% w/v) of sulfosalicylic acid. After filtration of the mixture, glacial acetic acid (2 mL), acid ninhydrin (2 mL) and filtrate (2 mL) were mixed. Then all the samples were kept at 100°C for 30 min using a water bath. After cooling, toluene (4 mL) were added and shaken it. Then, the samples were subjected to a spectrophotometer at 520 nm for measuring their optical densities.

### Leaf Glycinebetaine (GB) contents

The procedure outlined by Grieve and Grattan [32] was used to determine the GB contents in cauliflower leaf and inflorescence. Each of the dry samples were dipped in 10 mL of toluene (0.5%) and kept it overnight at 4°C and then centrifuged. After that, 1 mL of each of the extract and sulfuric acid (2N) were mixed. In a test tube, 0.5 mL supernatant was added and 0.2 mL of potassium tri-iodide solution (KI_3_) was also added to it. All the contents were mixed, cooled and added 1, 2 dichloroethane (5 mL) and distilled water (2.8 mL) to it. The absorbance of lower layer was noted at 365 nm using a spectrophotometer.

### Leaf hydrogen peroxide (H_2_O_2_)

Following Velikova et al. [33], each sample (0.25 mg) was chopped in 5 mL of TCA (0.1%). The extract was centrifuged and supernatant (0.5 mL) mixed in potassium iodide (KI; 1 mL) and potassium phosphate buffer (0.5 mL) and the absorbance was read at 390 nm.

### Malondialdehyde (MDA)

Lipid peroxidation was appraised by determining the development of malondialdehyde (MDA) with thiobarbituric acid (TBA). Leaf and inflorescence samples (each 0.25 g) were chopped each in 5 mL of 5% (w/v) trichloroacetic acid (TCA) using a mortar and pestle. After centrifugation, the supernatant was taken in a test tube and 4 mL of 0.5% (w/v) of TBA were added to 20% TCA. After cooling in an ice, the OD of the supernatant was noticed at 532 and 600 nm following Cakmak and Horst [34].

### Total phenolics

For the assessment of total phenolic contents, a procedure depicted by Julkunen-Tiitto [35] was followed. Fresh leaf and inflorescence tissues were chopped each in 3 mL acetone (80%) and the mixture was centrifuged at 10,000 × *g* for 10 min. In a test tube, 0.1 mL of the supernatant was taken and distilled water (2 mL) was added to it. Then 1 mL of the Folin-Ciocalteau’s phenol reagent was added. After shaking, 5 mL of 20% sodium carbonate were added to it and the volume was maintained up to 10 mL by using distilled water. A spectrophotometer was used to measure the absorbance of each treated sample at 750 nm.

### Ascorbic acid

Following Mukherjee and Choudhuri [36], the samples (each 0.25 g) were ground in 6% trichloroacetic acid (10 mL). Then, supernatant (4 mL) was mixed with 2 mL (2%) of dinitrophenyl hydrazine followed by one drop of thiourea. The mixture was placed in water bath for 15 min at 100°C and cooled. After it, H_2_SO_4_ (5 mL) were mixed and OD of each sample recorded at 530 nm.

### Enzymatic antioxidants

Plant leaf and inflorescence samples (0.25 g) were homogenized in 10 mL of 50 mM potassium phosphate buffer (pH 7.8) in a cooled pestle and mortar. After centrifugation the extract, the supernatant was used for the determination of the activities of catalase (CAT), superoxide dismutase (SOD) and peroxidase (POD) enzymes. Antioxidant enzyme activities were recorded on the basis of total soluble proteins assessed following Bradford [37].

### Activity of Catalase (CAT)

Following Luck [38], the activity of CAT enzyme was determined. The sample prepared by adding 0.1 mL of supernatant, 1 mL (5.9 mM) of hydrogen peroxide and 1.9 mL of 7.8 pH potassium phosphate buffer. The OD of samples were noted at 240 nm. The change in absorbance was recorded after every 20 s at 240 nm and change of 0.01 A_240_ unit min^–1^ was measured.

### Activity of Peroxidase (POD)

A protocol proposed by Chance and Maehly [39] was adopted to measure the activity of POD enzyme. The reaction mixture comprised of 0.1 mL extract, 1.9 mL (5.0 pH) potassium phosphate buffer, 100 μL of 20 mM guiacol and 1 mL (40 mM) of H_2_O_2_. Then, the absorbance was noted at 470 nm using a spectrophotometer.

### Activity of Superoxide Dismutase (SOD)

According to the procedure of van Rossum et al. [40], the enzyme extract (0.05 mL), NBT (50 μM), L-methionine (13 mM), potassium phosphate buffer (20 mM; pH, 7.8), triton-X, and riboflavin (1.3 μM) were mixed in a specific ratio. Then, the absorbance of the mixture was recorded at 560 nm using a spectrophotometer. One unit of SOD activity was defined as the amount required to inhibit the photo-reduction of NBT by 50%.

### Statistical analysis

ANOVA and the post hoc analysis of the data for all the attributes were employed using the CoSTAT statistical software. A least significant difference was calculated at 5% probability to compare the mean values for their significant differences.

## Results

### Growth characteristics

**Fig 1:**
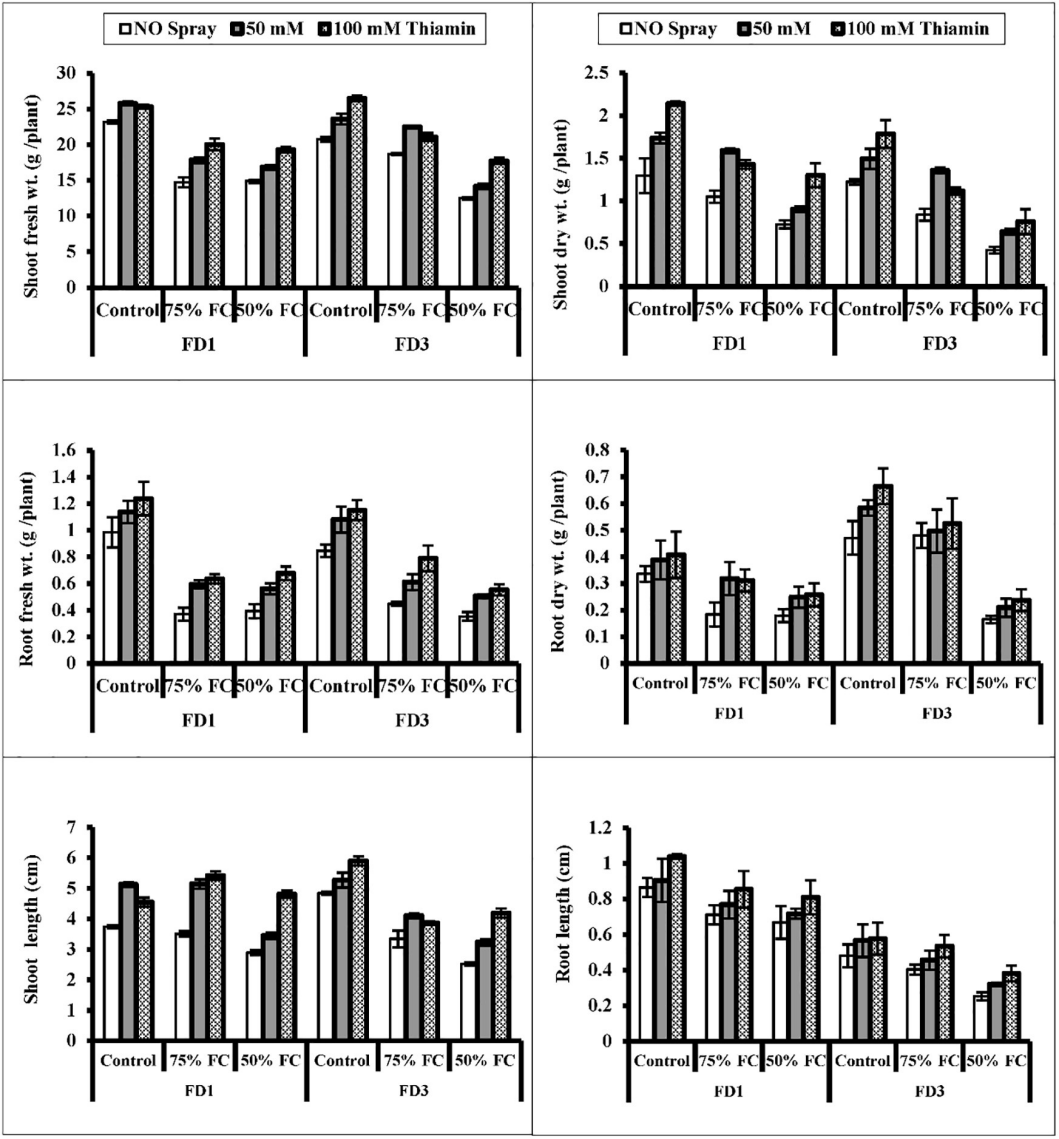
Shoot and root fresh and dry weights, shoot and root lengths of two cultivars of cauliflower (*Brassica oleracea* L.) plants foliarly treated with thiamin (50 and 100 mM) subjected to control (100% field capacity) and drought stress (75% and 50% field capacities). A significant (*P* ≤ 0.001) reduction in shoot and root lengths of cv. FD1 was observed at 50% drought level. While root length of cv. FD3 remained unchanged under both water regimes. Foliar-applied thiamin was effective (*P* ≤ 0.001) in improving the lengths (shoot and root) of both cauliflower cultivars as represented in Table 2. Of both cauliflower cultivars, cv. FD3 was lower while cv. FD1 higher in these attributes under water stress conditions (Fig 1).

Water stress regimes [75% and 50% field capacities (F.C.)] significantly (*P* ≤ 0.001) suppressed the growth in terms of root and shoot weights (fresh and dry) of both cultivars (FD3 and FD1) of cauliflower. However, foliar-applied thiamin (50 and 100 mM) caused an improvement in the above-mentioned growth attributes of both cauliflower cultivars, particularly 100 mM of thiamin was found to be very effective under water deficit regimes (Fig 1; Table 1). To assess the difference between cultivars with respect to drought stress and thiamin treatments, the post hoc analysis was carried out. The post hoc analysis indicated that cauliflower cultivar, FD3 was not affected significantly in root fresh weight at both 75% and 50% and root dry weight at 75% field capacities (Table 2). Post hoc analysis for shoot fresh and dry weights and root fresh weight of both cauliflower cultivars showed improvement at both levels of thiamin. While root dry weight of cv. FD3 remained unchanged by exogenous application of thiamin (Table 2).

**Table 1:** Mean square values for growth and leaf biochemical characteristics of cauliflower (*Brassica oleracea* L.) plants treated with thiamin subjected to water deficit stress.

Source of variation	df	Shoot fresh weight	Shoot dry weight	Root fresh weight	Root dry weight	Shoot length	Root length
Cultivars (Cvs)	1	0.021ns	0.92***	0.31***	0.07ns	0.322*	2.236***
Drought (D)	2	447.0***	3.07***	1.636***	0.426***	12.6***	0.356***
Thiamin (T)	2	123.5 ***	2.788***	2.75***	0.91***	11.9***	1.61***
Cvs x D	2	40.2***	0.12***	0.051*	0.09*	6.154***	0.062***
Cvs x T	2	0.498ns	0.048*	0.102***	0.332***	0.467***	0.163***
D x T	4	2.93**	0.403***	0.324***	0.041ns	0.84***	0.046***
Cvs x D x T	4	11.27***	0.033*	0.126***	0.067*	0.811***	0.113***
Error	54	0.636	0.010	0.011	0.024	0.057	0.004
		**Chl. a**	**Chl. *b***	**Chl. *a*/*b* ratio**	**Total chlorophyll**	**RMP**	**Proline**
Cultivars (Cvs)	1	0.029ns	0.045*	2.160*	0.001ns	888.9ns	0.365*
Drought (D)	2	0.024ns	0.1007***	2.806***	0.1003ns	107.1ns	3.91***
Thiamin (T)	2	0.1703**	0.031*	1.945**	0.253**	2436.3*	2.434***
Cvs x D	2	0.011ns	0.011ns	0.129ns	0.043ns	819.6ns	0.35**
Cvs x T	2	0.010ns	0.003ns	0.038ns	0.024ns	417.5ns	0.149ns
D x T	4	0.048ns	0.001ns	0.47ns	0.055ns	200.01ns	0.173*
Cvs x D x T	4	0.005ns	5.586ns	0.050ns	0.003ns	244.8ns	0.21**
Error	54	0.027	0.006	0.344	0.043	563.77ns	0.051
		**GB**	**Total soluble Proteins**	**Ascorbic acid**	**MDA**	**H**_**2**_ **O**_**2**_	**Total phenolics**
Cultivars (Cvs)	1	765.6***	1800.002ns	326.13***	12.65ns	0.407ns	34.13***
Drought (D)	2	3487.4***	51758.6***	175.75***	142.76***	36.32***	150.6***
Thiamin (T)	2	1556.6***	153230.6***	58.28***	78.42***	7.638***	333.3***
Cvs x D	2	730.5***	22999.9***	25.45**	54.33***	4.86**	36.44***
Cvs x T	2	165.8***	5930.3*	1.49ns	3.98ns	0.551ns	4.078ns
D x T	4	249.4***	6624.22**	2.403ns	3.698ns	0.737ns	4.23ns
Cvs x D x T	4	86.4**	3449.2ns	1.288ns	6.254ns	0.368ns	3.19ns
Error	54	20.44	1629.02	3.728	5.507	0.855	2.565
		**SOD**	**CAT**	**POD**			
Cultivars (Cvs)	1	0.799ns	0.414ns	126.78ns			
Drought (D)	2	44.64***	3.960***	467.20***			
Thiamin (T)	2	57.67***	5.99***	449.62***			
Cvs x D	2	3.481ns	1.645*	1.632ns			
Cvs x T	2	1.442ns	0.018ns	2.64ns			
D x T	4	2.786ns	0.210ns	18.65ns			
Cvs x D x T	4	1.042ns	0.0917ns	1.973ns			
Error	54	2.934	0.351	31.71			

Chl, Chlorophyll; RMP, Relative Membrane Permeability; GB, Glycinebetaine; MDA, Malondialdehyde; H_2_O_2_, Hydrogen peroxide; SOD, Superoxide dismutase, CAT, Catalase; POD, Peroxidase

**Table 2:** Mean square values (post-hoc analysis) of different growth and biochemical attributes of cauliflower plants treated with thiamin under water deficit stress.

Parameters	Cultivars	Drought		Thiamin		Parameters	Cultivars	Drought		Thiamin	
		75% F.C.	50% F.C.	50 mM	100 mM			75% F.C.	50% F.C.	50 mM	100 mM
Shoot fresh weight	FD1	143.1***	138.0***	13.23***	8.85**	Shoot dry weight	FD1	0.0001ns	0.21**	0.95***	2.4***
	FD3	8.68**	136.7***	28.5***	82.44***		FD3	0.293**	1.44***	0.54***	1.08***
Root fresh weight	FD1	0.48***	0.43***	0.81***	0.285*	Root dry weight	FD1	0.007*	0.001ns	0.81***	0.862**
	FD3	0.00001ns	0.003ns	0.48**	1.697***		FD3	0.028ns	0.378*	0.001ns	0.047ns
Shoot length	FD1	0.116ns	1.45***	3.81***	1.301**	Root length	FD1	0.02ns	0.22***	0.57***	0.46***
	FD3	4.47**	10.78***	1.141*	3.079***		FD3	0.012ns	0.011ns	0.548***	0.453***
Chlorophyll *a*	FD1	0.016ns	0.01ns	0.014**	0.012ns	Chlorophyll *b*	FD1	0.026*	0.02*	0.001ns	0.006ns
	FD3	0.019ns	0.00001ns	0.26**	0.339ns		FD3	0.006ns	0.021*	0.009ns	0.23ns
Chlorophyll *a/b* ratio	FD1	0.04ns	1.777ns	0.04ns	0.0008ns	Total chlorophyll	FD1	0.08ns	0.0006ns	0.024*	0.036ns
	FD3	0.054ns	0.454ns	2.04ns	2.577ns		FD3	0.048ns	0.023ns	0.369*	0.112ns
Proline	FD1	0.21*	1.51***	0.318*	0.417**	Glycinebetaine	FD1	340.8**	34.33ns	0.106ns	19.45ns
	FD3	0.094*	0.002ns	0.074**	0.281ns		FD3	314.3***	817.5***	302.4*	130.2**
Relative membrane permeability	FD1	75.7ns	98.21ns	381.7ns	248.2ns	Ascorbic acid	FD1	35.52**	98.09***	0.78ns	18.16*
	FD3	350.6ns	154.7ns	3248.9*	508.3ns		FD3	7.08ns	6.07**	4.55ns	8.186**
Malondialdehyde	FD1	17.01ns	162.2**	14.22ns	7.44ns	Hydrogen peroxide	FD1	0.168ns	8.10**	0.804ns	1.97*
	FD3	30.58*	11.68ns	38.78*	14.91*		FD3	11.65**	15.5**	3.19ns	0.223ns
Total soluble proteins	FD1	26026**	1152.2ns	6763.1*	30432.9**	Total phenolics	FD1	18.83*	41.51**	10.12*	61.74**
	FD3	3253.7ns	5560.7ns	4374.5ns	22892**		FD3	12.68*	86.79***	18.37*	104***
Superoxide dismutase	FD1	1.34ns	23.1*	8.78ns	16.72ns	Peroxidase	FD1	32.29ns	281.9***	157.8*	200.9*
	FD3	0.008ns	2.57ns	2.03ns	43.07**		FD3	28.49ns	215.4**	20.82ns	176.1**
Catalase	FD1	0.273ns	2.62*	0.663ns	1.443*						
	FD3	0.07ns	0.02ns	0.884ns	1.438*						

### Leaf physio-biochemical characteristics

**Fig 2:**
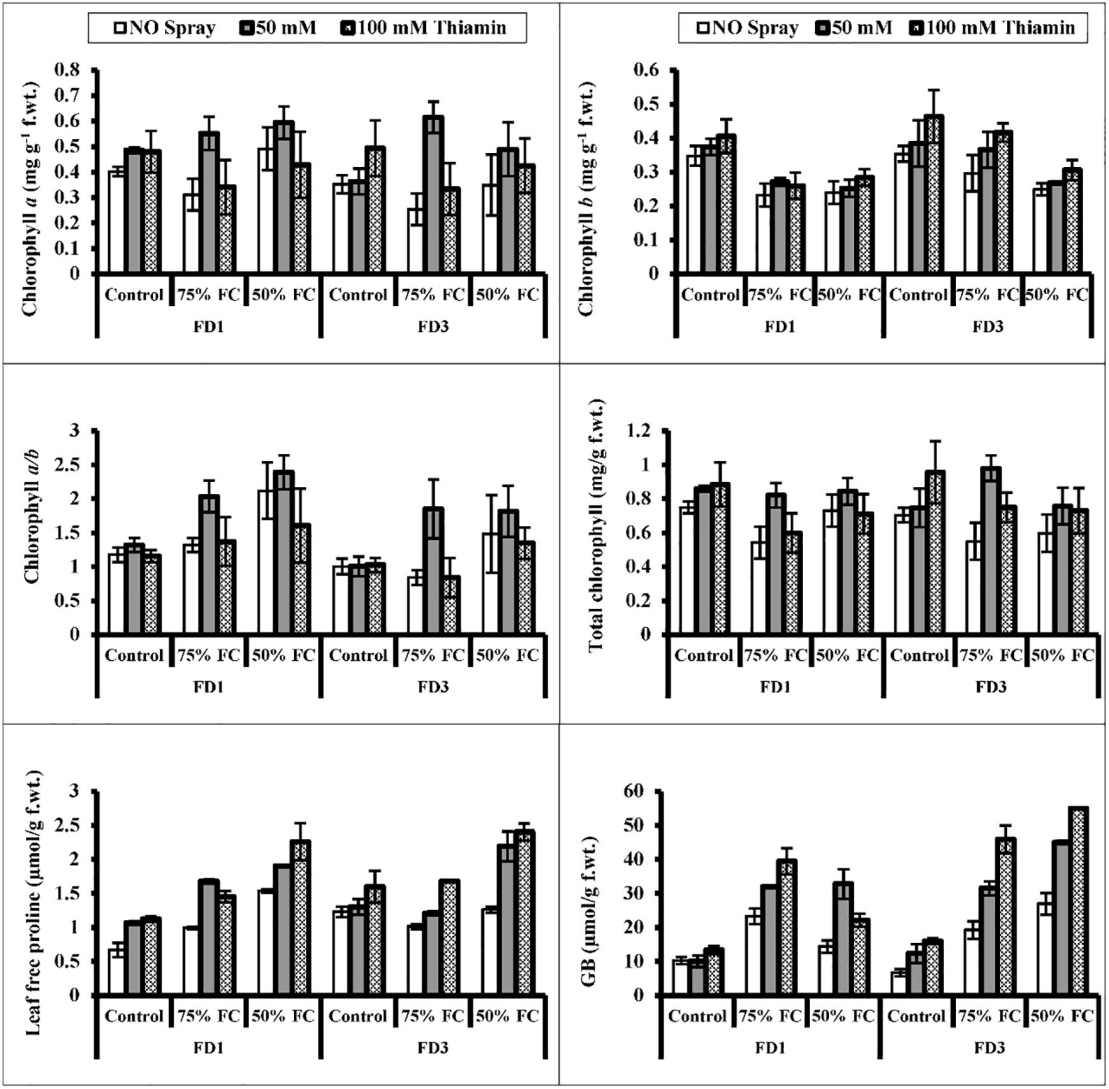
Leaf chlorophyll *a*, *b*, *a/b*, total chlorophyll, proline and Glycinebetaine (GB) contents of two cultivars of cauliflower (*Brassica oleracea* L.) plants foliarly treated with thiamin (50 and 100 mM) subjected to control (100% field capacity) and drought stress (75% and 50% field capacities). A significant increase in leaf proline and GB contents in both cauliflower cultivars was observed particularly at 75% drought stress conditions. Exogenously applied thiamin (50 mM) had a positive effect in enhancing the accumulation of proline in both cauliflower cultivars under water-deficit regimes. Post-hoc analysis showed that cv. FD3 was better in GB accumulation due to exogenously applied thiamin under water-stressed conditions (Fig 2; Table 2).

Water stress (75% and 50% F.C.) had a non-significant effect on chlorophyll (*a* and total) contents and chlorophyll *a/b* ratio of both cauliflower cultivars (Tables 1 & 2). While a significant (*P* ≤ 0.05) reduction in chlorophyll *b* was observed in both cauliflower cultivars at 50% F.C. Foliar-applied 50 mM of thiamin was effective in improving the total and chlorophyll *a* concentration in both (FD1 and FD3) cauliflower cultivars under non-stress and water stress conditions (Fig 2). Of both cauliflower cultivars, cv. FD3 was better in chlorophyll *b* contents under all water regimes.

**Fig 3:**
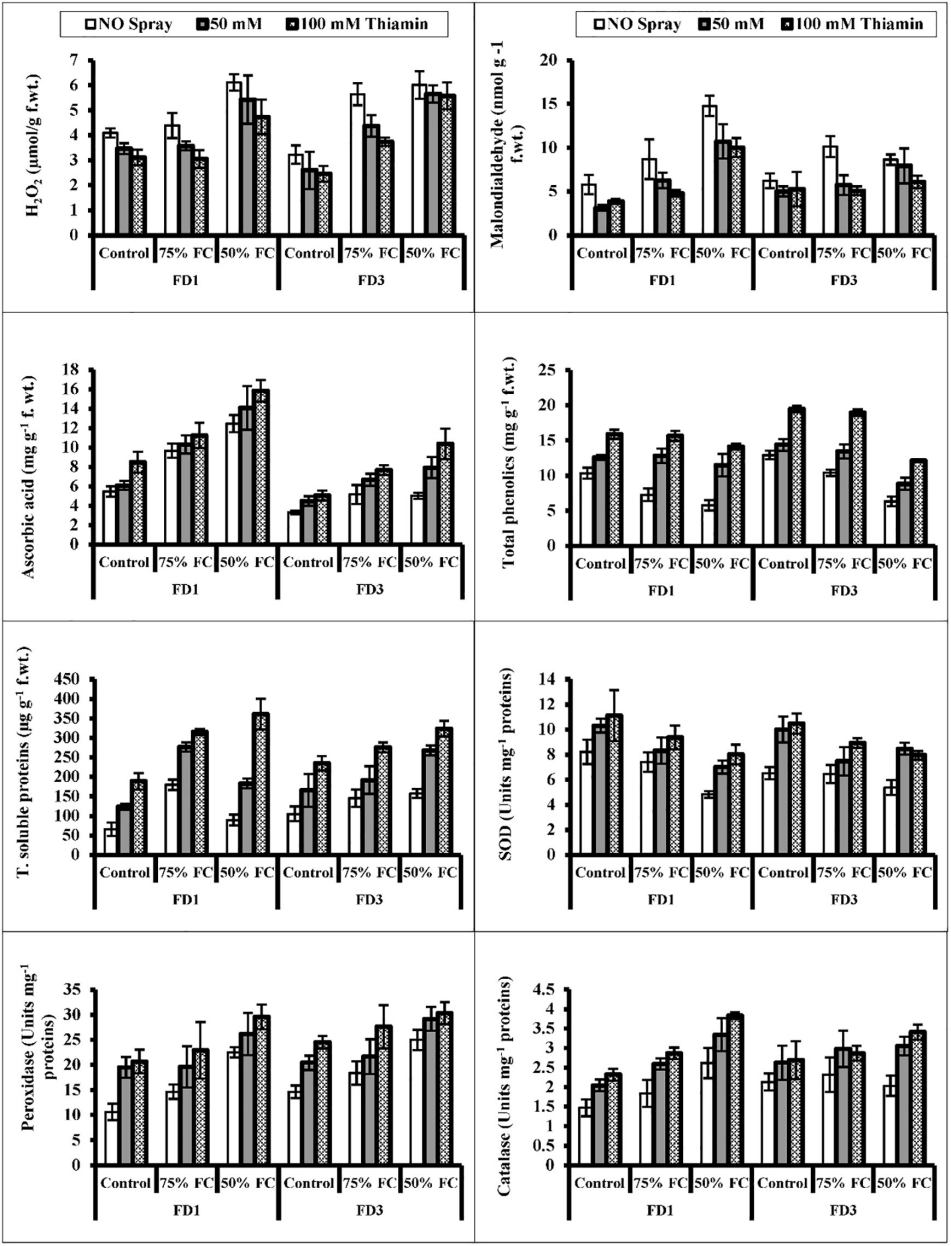
Leaf hydrogen peroxide, MDA, ascorbic acid, total phenolics, leaf total soluble, activities of peroxidase, superoxide dismutase and catalase enzymes of two cultivars of cauliflower (*Brassica oleracea* L.) plants foliarly treated with thiamin (50 and 100 mM) subjected to control (100% field capacity) and drought stress (75% and 50% field capacities). Accumulation of ascorbic acid (AsA) increased significantly (*P* ≤ 0.001; 0.01) particularly at 50% F.C. of both cauliflower cultivars. However, foliar applied thiamin (100 mM; *P* ≤ 0.001) increased AsA contents in both cauliflower cultivars under varying water regimes. A marked difference was noted in both cultivars in terms of AsA contents, and cv. FD1 was significantly better that the other cultivar in AsA contents at varying thiamin and water stress levels (Fig 3).

Water deficit conditions significantly enhanced (*P* ≤ 0.001) the accumulation of MDA in cv. FD1 at 50% F.C. and in cv. FD3 at 75% F.C. and H_2_O_2_ contents in both cauliflower cultivars particularly at 50% F.C. Foliar-applied thiamin (50 and 100 mM) was considerably effective in decreasing the MDA in cv. FD3 and H_2_O_2_ contents in cv. FD1 only at 100 mM under both water regimes (Table 2; Fig 3).

Total phenolics affected significantly in both cauliflower cultivars under water deficit conditions. Exogenously applied thiamin was very effective in increasing the total phenolics in both cauliflower cultivars. A maximum increase was observed at 100 mM of thiamin in cv. FD3 at 75% F.C. (Fig 3).

The post hoc analysis indicated that total soluble proteins increased significantly (*P* ≤ 0.01) only in cv. FD1 at 75% water deficit conditions. However, exogenously applied both levels of thiamin were effective (*P* ≤ 0.05 and 0.01) in increasing the total soluble proteins contents in cv. FD1 and 100 mM in cv. FD3 under stress conditions. A maximum increase was observed at 100 mM of thiamin at 50% F.C. in both cauliflower cultivars (Fig 3).

The activity of superoxide dismutase (SOD) enzyme was found to be decreased and the activity of catalase (CAT) increased only in cv. FD1 at 50% F.C. An increase was observed in the activity of peroxidase (POD) enzyme at 50% F.C. in both cauliflower cultivars (Fig 3; Table 2). Thiamin (100 mM) considerably upregulated the activities of leaf SOD in cv. FD3, the activities of CAT and POD enzymes at 100 mM in both cauliflower cultivars under varying water regimes (Table 2).

### Biochemical characteristics of inflorescense

**Fig 4:**
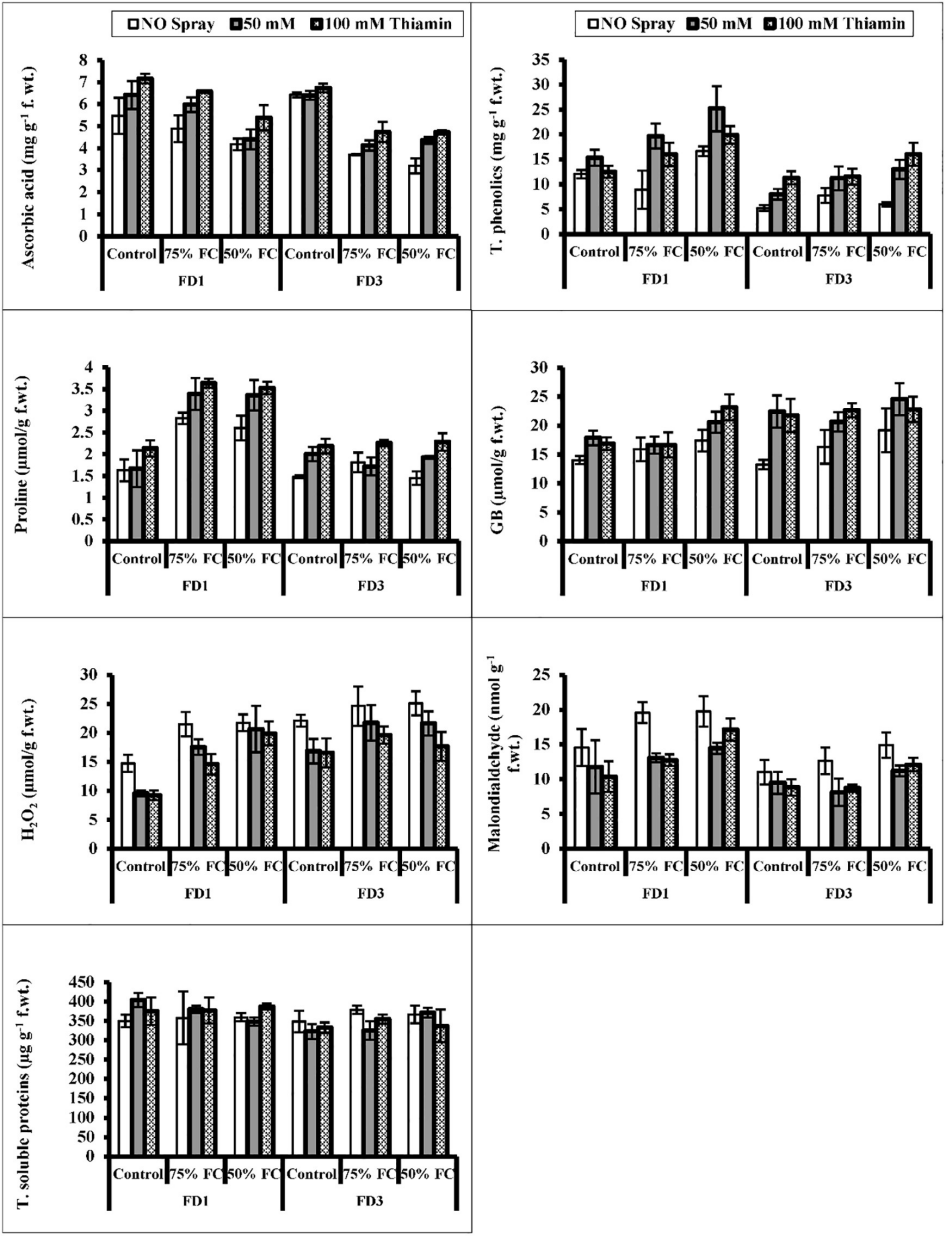
Ascorbic acid, total phenolics, proline, GB, hydrogen peroxide, MDA and total soluble proteins in the inflorescence of two cultivars of cauliflower (*Brassica oleracea* L.) plants foliarly treated with thiamin (50 and 100 mM) subjected to control (100% field capacity) and drought stress (75% and 50% field capacities). A significant increase in inflorescence proline and GB contents was observed under water-deficit conditions (Fig 4). Exogenous application of thiamin increased (*P* ≤ 0.001) the contents of GB and proline in both cauliflower cultivars under varying water regimes. A maximum increase in GB and proline contents was recorded in cauliflower plants at 50% F.C.

Inflorescence analyses showed that water deficit conditions decreased the AsA contents, while an increase in the accumulation of total phenolics was observed under water deficit conditions (Fig 4). A significant enhancement (*P* ≤ 0.001) was observed in total phenolics and AsA concentrations of both cauliflower cultivars under foliar-applied thiamin under water stress. Overall, cv. FD1 showed good response as compared to cv. FD3 in these attributes, particularly under drought stress conditions.

Under water deficit regimes, a considerable increase was observed in MDA and H_2_O_2_ contents in the inflorescence of both cauliflower cultivars. However, application of thiamin effectively reduced the head MDA and H_2_O_2_ accumulation under water stress conditions. Of both cauliflower cultivars, cv. FD3 was higher in H_2_O_2_ and cv. FD1 in MDA contents under water deficit regimes (Fig 4).

A non-significant effect of water stress as well as thiamin application was observed on the total soluble proteins of inflorescence of cauliflower plants subjected to varying water stress conditions (Fig 4). The response of both cauliflower cultivars to thiamin and water stress treatments also remained constant with reference to inflorescence total soluble proteins.

**Fig 5:**
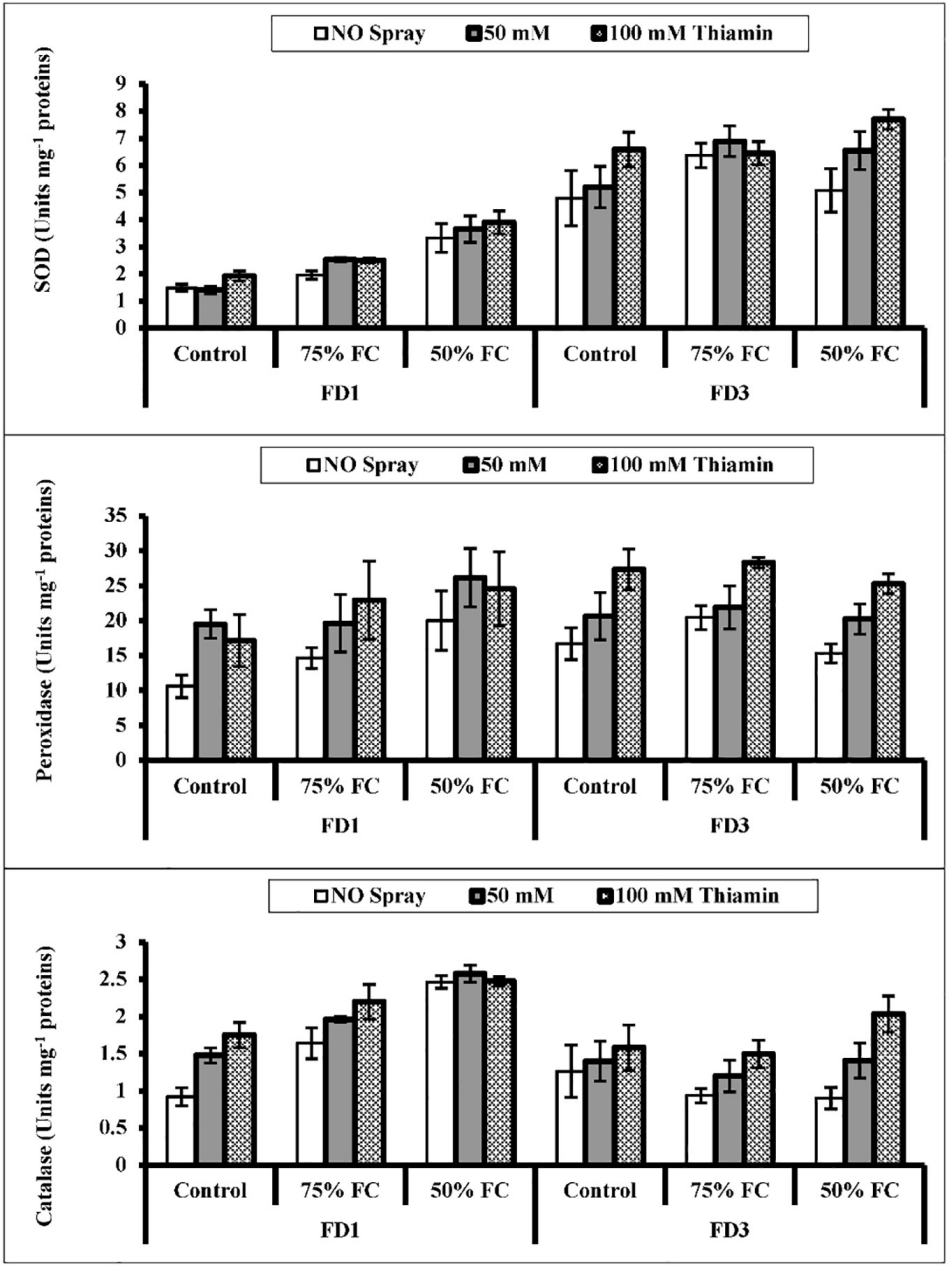
Activities of Superoxide Dismutase (SOD), Catalase (CAT) and Peroxidase (POD) enzymes in the inflorescence of two cultivars of cauliflower (*Brassica oleracea* L.) plants foliarly treated with thiamin (50 and 100 mM) subjected to control (100% field capacity) and drought stress (75% and 50% field capacities).

A remarkable (*P* ≤ 0.001) upregulation in the activities of SOD and CAT enzymes was observed due to water-deficit conditions in the inflorescence of both cauliflower cultivars. Foliage spray with thiamin improved (*P* ≤ 0.001) the activities of SOD, POD and CAT enzymes under water stress and non-stress conditions (Fig 5). Of both cauliflower cultivars, cv. FD3 was superior to the other cultivar in the activity of the inflorescence SOD enzyme, while the reverse was true in cv. FD1 with reference to the CAT activity. However, the response of both cauliflower cultivars was almost similar in terms of the POD activity under varying water regimes.

## Discussion

Water stress is a vital ecological factor adversely affecting plant growth, yield and metabolism involved therein [5, 41, 42]. Plants, being sessile living entity, can face water deficiency at every stage of plant growth which can effectively impair physio-biochemical processes, e.g., stomatal regulation, cell growth (division and elongation), photosynthetic rate, water relations, hormonal regulation and nutrients’ uptake and utilization [43, 44]. In the current study, drought stress significantly reduced the shoot and root weights of both cauliflower cultivars (FD3 and FD1) at 50% F.C. However, thiamin (vitamin B1) significantly improved the plant growth under varying water regimes. Of different plant growth bio-regulators, thiamin could be considered as a co-factor, because it plays a vital role in ameliorating the adversaries of abiotic stresses in plants [17, 20, 45]. Application of thiamin proved beneficial in enhancing drought stress tolerance of turnip [45] and alfalfa [19] plants, while salinity tolerance of sunflower [24] and maize [20] plants. Moreover, under non-stress conditions, Aminifard, Jorkesh [46] found that foliar-applied thiamin particularly at the rate of 500 mg L^-1^ stimulated the growth, yield and biochemical (nitrogen, phosphorus, carotenoid and chlorophyll *b*) compounds of coriander and fenugreek plants. Recently, Jabeen et al. [45] found that thiamin improved the plant growth and yield of turnip plants under water-deficit conditions (75% and 50% field capacity). They found that thiamin-induced improvement in turnip plants to increased photosynthetic pigments, antioxidant capacity, accumulation of proline and GB and suppressed ROS (H_2_O_2_ contents) under drought stress. Under drought stress, the activation of osmotic adjustment has been well determined phenomenon to tolerate water deficiency in plants. Under this condition, osmoregulation, ion homeostasis as well ionic compartmentalization can be involved and well reported. In the present study, the upregulation of proline, GB as well as oxidative defense system might be involved in the drought stress tolerance of cauliflower plants.

Chlorophyll pigments play an important role in light harvesting and energy dissipation and are also main constituents of chloroplast which regulate the rate as well as the process of photosynthesis under stress and controlled conditions [47]. Photosynthesis and chlorophyll pigments are believed to be reduced due to ROS over-accumulation as a result of oxidative stress under drought stress [41, 48]. In this study, chlorophyll *b* contents reduced in both cauliflower cultivars at 50% F.C. But the foliar treatment with thiamin enhanced the chlorophyll (*a* and *b*) contents in both cultivars of cauliflower. Analogous to our results, Ghaffar, et al. [19] reported that chlorophyll content decreased due to the shortage of water, but thiamin (100 mM) enhanced the chlorophyll content in white clover cultivars. It is well reported that thiamin is effective in minimizing the production of ROS and improving oxidative defense system, which might be involved in protecting the chloroplast and its pigments from the adversaries of abiotic stresses including water deficiency [19, 49].

An increase in ROS was observed on exposure to drought stress in different plant species such as maize [5], quinoa [50], turnip [45], and broccoli [51], etc. In this trial, H_2_O_2_ and MDA (an indicator of lipid peroxidation) found to be improved in the leaf as well as head of both cauliflower cultivars emulating the overexpression of lipid peroxidation and ROS in the water-stressed cauliflower plants. Drought-induced increase in over-production of H_2_O_2_ and MDA has already noticed in canola [41], cucumber [52], and wheat [53] etc. However, foliar applied thiamin was effective in reducing the accumulation of MDA and H_2_O_2_ contents in the leaf as well as head of both cauliflower cultivars at 50% F.C. Similarly, Kaya, et al. [20] observed that thiamin decreased the accumulation of MDA and H_2_O_2_ concentrations in salt-stressed maize plants. Moreover, Sayed and Gadallah [24] reported that exogenous application of thiamin either sprayed on shoots or applied via roots, minimized the harmful effects of salt stress on growth of sunflower plants by minimizing oxidative stress and upregulating anti-oxidative system. In addition, thiamin-treated maize plants were better in relative water content, amino acids and soluble sugars under saline conditions. It has also been reported that thiamin treatment increased plant tolerance to parquet through the suppression of ROS production and consequently oxidative damage [54]. Overall, this data indicated that modulation of thiamin metabolism is important to maintain the normal functioning of plants subjected to abiotic stresses including drought stress. So, a possible role of thiamin in signaling as an adaptation mechanism has been suggested [17, 45].

Osmotic adjustment is one of the important phenomena to maintain stomatal conductance, leaf water status and water potential in plants to maintain balanced growth under drought stress. Maximum production of osmoprotectants particularly GB and proline protects the plants under stress conditions by upregulating enzyme metabolism, membrane stability and ROS detoxification under water scarcity [55, 56]. We observed that compatible osmolytes (GB and proline) significantly increased in both leaf and head of cauliflower cultivars under water-deficit stress. In general, high accumulation of GB and proline has already been reported to be associated with drought stress tolerance [50, 57]. For example, better growth of drought stressed maize plants has already been associated with higher levels of GB and proline accumulation, one of the important aspects of osmotic adjustment under stress conditions [5].

The role of thiamin in plant stress tolerance has been explored but the sole mechanism (s) underlying still debatable [58]. In recent times, it was noticed that thiamin plays a defensive role in plants on exposure to abiotic stresses. Application of thiamin neutralized damaging effects of environmental stresses on plants [5]. Previously, it has been reported that thiamin participates in the underlying processes of stress-induced oxidative stress [17]. For example, Rapala-Kozik et al. [22] observed the involvement of a number of genes in the synthesis of enzymes regulating the biosynthesis of thiamin particularly in *Arabidopsis thaliana*. Of various non-enzymatic antioxidants, total phenolics and ascorbic acid contents are the most important substances that help in the detoxification of reactive oxygen species [5]. In the present study, total phenolics and AsA in head of both cauliflower cultivars were increased due to foliar-applied thiamin under drought stress conditions. In another study with carrot plants, an increase in tissue total phenolics was observed on exposure to drought stress conditions [59]. Likewise, Ghaffar, et al. [19] reported an increase in the ascorbic acid contents due to foliar applied thiamin in water-stressed white clover plants which in turn promote growth of these plants under stress conditions. High accumulation of AsA might be associated with drought stress tolerance by upregulation the oxidative defense system.

Enzymatic oxidative defense system such as up-regulation of the activities of POD, SOD, CAT and glutathione reductase enzymes is also believed to be involved in better plant growth and yield production under stress conditions [15, 60]. Of all enzymatic antioxidants, the over-generation of SOD enzyme plays an important role in catalyzing the detachment of two molecules of superoxide to give rise O_2_ and H_2_O_2_. While, catalase and peroxidase enzymes work jointly to stabilize the quantity of latent oxidants, thereby recovering the plant tolerance to water deficiency [15, 61]. In the present study, foliar-applied thiamin upregulated the activity of CAT and SOD enzymes in both cultivars of cauliflower under non-stress and stress conditions. In an earlier study, Kaya, et al. [20] found that foliar applied thiamin improved the antioxidant potential of maize plants under saline stress conditions. Moreover, Mansouri, et al. [62] reported that thiamin treatment was also effective in improving the antioxidant capacity of *Gerbera jamesonii* plants.

Like several other vegetables, cauliflower accumulates a variety of nutrients and metabolites in their leaves and head. The leaves have been reported to contain significant amounts of dietary fiber, Fe, K, Ca, essential fatty acids, β-carotenes, folate and a myriad of antioxidant biomolecules. Moreover, its head is rich in vitamin C and K as well as folate [63]. Like in cauliflower leaves, the head also contains significant amounts of phytochemicals and antioxidants [64]. However, the fresh appearance of cauliflower’s head and its reasonably promising nutrition levels have been reported to be attributable to plant part water holding capacity and translocation of essential mineral elements including primarily N [65, 66]. In fact, N plays a vital role in the synthesis of a variety of amino acids, vitamins and proteins and thus it determines the yield and quality of the cauliflower crop [65, 67, 68]. Like several other inorganic nutrients, an optimal N translocation from the shoot (source) to the head (sink) is believed to determine nutritional quality of cauliflower [65, 68, 69]. It is now evident that metabolites transport particularly of sugars and carbohydrates between cells, organelles as well as source and sink tissues is well coordinated, and it facilitates to gather information related to resource and its utilization [70]. However, little information is available in the literature on the transport of metabolites investigated from the source to the sink in cauliflower. Thus, this certainly needs to be explored in the future studies. However, it is amply clear that metabolites involved in the signaling mechanism may ensure resource allocation to drive better growth and development under stress and non-stress conditions. Moreover, information on the concentration and composition of metabolites during fruit development and maturity can help understand their regulation during these processes for achieving better fruit quality [71].

## Conclusions

In conclusion, drought stress decreased plant growth, leaf total phenolics, the activity of SOD enzyme as well as inflorescence AsA contents, while increased leaf proline, GB, MDA, H_2_O_2_, AsA, total soluble proteins, the activities of POD and CAT enzymes along with inflorescence total phenolics, proline, GB, MDA, H_2_O_2_, activities of SOD and CAT enzymes in both cauliflower cultivars. However, foliar-applied thiamin enhanced plant growth, photosynthetic pigments, GB, proline, AsA, total phenolics, total soluble proteins and the activities of CAT, POD and SOD in both cauliflower cultivars under water stress regimes. In addition, foliar applied thiamin was effective in reducing the leaf and inflorescence MDA and H_2_O_2_ contents of cauliflower. Overall, exogenously applied thiamin was effective in improving the plant growth particularly at 50% F.C. which was found to be ascribed with better osmoregulation as well as oxidative defense system and a significant suppression in the production of ROS in cauliflower plants.
